# Public involvement could usefully inform ethical review, but rarely does: what are the implications?

**DOI:** 10.1186/s40900-017-0080-0

**Published:** 2017-12-11

**Authors:** Kristina Staley, Jim Elliott

**Affiliations:** 1 TwoCan Associates, Montague House, 4 St. Mary’s Street, Ross on Wye, HR9 5HT UK; 2 Public Involvement Lead, Health Research Authority, Ground Floor, Skipton House, 80 London Road, London, SE1 6LH UK

**Keywords:** Public involvement, Patient involvement, Research ethics, Ethical review

## Abstract

**Plain English summary:**

Researchers carrying out research in the NHS in England have to obtain approval for their study from an NHS Research Ethics Committee (REC). Involving the public in research helps to ensure studies are ethically acceptable to the people taking part, and therefore supports the REC review. The form used by RECs asks researchers to describe any involvement that has taken place before the review or any planned for the future. We analysed researchers’ reports of involvement in 2748 applications to RECs in 2014, to assess how well their approaches to involvement are informing the review process. We found that researchers rarely describe involvement in enough detail to help REC members. It is difficult to judge whether previous involvement has shaped the research design in any way, and whether plans for future involvement are meaningful. It also seems that some researchers remain unclear about involvement and its purpose at different stages. This may be severely limiting its impact.

So that public involvement can usefully inform REC reviews in future, the Health Research Authority, which oversees RECs, will carry out further work to find out what information RECS need about involvement. This information will be used to change the application form and to develop guidance and training for REC members and the wider research community. Researchers may also benefit from clearer guidance on the value and purpose of involvement at key research stages: early design, data collection and the dissemination of results.

**Abstract:**

**Background** Researchers conducting research in the NHS in England are required to submit their study for approval by an NHS Research Ethics Committee (REC). Public involvement in research prior to REC review helps to ensure studies are ethically acceptable to participants, thus informing the review process. The Integrated Research Application System (IRAS) used by RECs, asks researchers to describe any involvement in the development of their project, and in its delivery and dissemination. We analysed researchers’ reports of involvement to assess how well current approaches to involvement are supporting REC review.

**Methods** We used a mixed methods approach. The anonymised free-text data from all 2748 non-educational applications submitted to RECs in 2014 were analysed using NVivo. Themes were developed from the data and used to summarise and categorise the different types of reports of involvement. The frequency of common types of report was analysed using simple statistics.

**Results** In general, researchers rarely describe any prior involvement in sufficient detail to know what was done and what difference this made. This makes it difficult to judge whether the involvement shaped the research design in any way to make it more ethically acceptable. Similarly, researchers’ plans for future involvement are not clear enough to enable RECs to make a proper assessment of whether this involvement will be meaningful, or whether potential ethical concerns raised by involvement have been addressed. This analysis also shows there is still considerable misunderstanding amongst researchers around what involvement means, and its purpose at different stages of a project. This may be severely limiting the potential for impact.

**Conclusions** So that public involvement can usefully inform REC review in future, the HRA is undertaking a collaborative exercise to understand what information RECS need about involvement, and what changes need to be made to the IRAS form. At the same time it will develop guidance and training for REC members and the wider research community about how public involvement can support ethical review. Researchers may also benefit from guidance on the value and purpose of involvement at the research stages: design, data collection and dissemination of results.

## Background

Researchers conducting most types of research in the NHS in England are required to submit their study for ethical review by an NHS Research Ethics Committee (REC) within the Health Research Authority’s Research Ethics Service (RES) [1]. Involving the public^1^ in research prior to REC review helps to design studies that are ethically acceptable to the people who will be taking part [2–6]. Public involvement addresses the issues of main concern to REC decision-making [7], by helping to ensure that:

- the research genuinely reflects the interests of the people who will potentially benefit
- the study design is ethically acceptable to participants and their practical and support needs will be met
- the process of obtaining consent genuinely informs potential participants
- the findings will be communicated to participants and the wider public [3, 4]

**Fig. 1:**
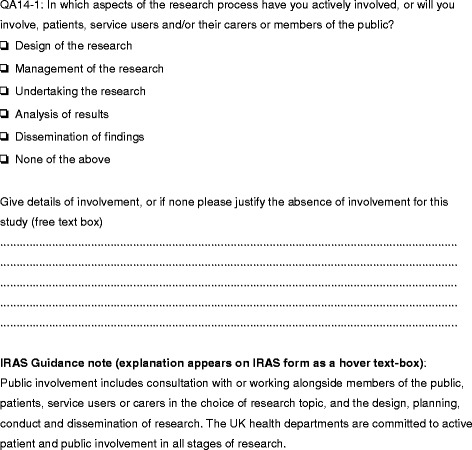
The question on the IRAS form that asks researchers about public involvement in their study

In recognition of the importance of public involvement in helping to ensure the ethical acceptability of research, the Integrated Research Application System (IRAS) application form includes a question (QA14-1) about public involvement (see Fig. 1). This question asks researchers to describe how the public have contributed to the planning and design of the proposed research and how they will continue to be involved in its conduct and management. It contains a series of tick-boxes for researchers to identify which stages of the research cycle they have involved or plan to involve the public (design, management, undertaking, analysis and/or dissemination), plus a free-text box where they can describe the involvement in detail, or justify their decision to have no involvement in their research.

Since 2010, a joint Health Research Authority (HRA) and INVOLVE study has, on a two-yearly basis, analysed the extent of public involvement in applications for ethical approval assessed by RECs [8–10]. These analyses have shown an increase in the overall proportion of applications reporting some form of public involvement at one or more stages of the research cycle. In 2014, 36% of studies reported involvement, up from 28% in 2012 and 19% in 2010 [10]. (A more detailed breakdown of these figures is available in reference [10]). While the analysis to date has been mainly focused on whether researchers report any kind of involvement in research in their IRAS applications, for this article, we undertook additional qualitative and quantitative analyses to explore what types of involvement were being reported, how often and with what impact. By analysing researchers’ free-text responses in 2014, we aimed to address the following questions:

- How are researchers involving the public *prior* to ethical review and how well does this support the review process?
- How are researchers planning to involve the public in the *future* delivery of research and what are the implications for ethical review?

Our overall aim was to gather evidence to support REC members in their interpretation of researchers’ reports of involvement, and to gain greater insights into the link between involvement and REC decision-making. However, our analysis showed that currently public involvement in research is not reaching its full potential to inform ethical review. There appear to be two main problems. Firstly, the information that researchers provide about involvement is often very limited in detail, making it difficult to know precisely what difference the involvement made. Secondly, in some researchers’ descriptions of public involvement, there seem to be some common misunderstandings about what involvement looks like at different stages of research and lack of clarity about what to report to RECs. This article describes these issues in more depth, and discusses the implications for the HRA and for involvement practice.

## Methods

The anonymised data^2^ from responses to question QA14-1 from all 2748 non-educational^3^ applications submitted to the HRA RES in 2014 were included in this analysis, which took place in two phases as described below. When researchers report on involvement in the application form, they tick a box to indicate at which stage(s) involvement took place or is planned, and then describe this involvement in free-text. The free-text responses vary from being one sentence to several paragraphs. Our analysis of the responses on these forms combined both qualitative and quantitative approaches using Excel and NVivo.

### Phase 1

Initially we selected all the applications where the researcher had ticked at least one box to report that some kind of involvement had already taken place or was planned (*n* = 1731). We reviewed all the free-text responses within these applications to see if they contained any description of involvement that reflected INVOLVE’s definition.^4^ We were generous in this interpretation, so that even if a researcher simply stated that patients/the public had been involved, this was considered as indicating involvement had taken place. JE and KS each analysed half of these responses and both analysed an overlapping sample (*n* = 201) to check we were applying INVOLVE’s definition in the same way. We used Cohen’s kappa co-efficient [11], a measure of agreement between two raters, to assess the consistency of our approach. This confirmed a high level of agreement (our score was 0.87 and a value greater than 0.80 indicates very good agreement). Where there was disagreement on whether the reported involvement did reflect INVOLVE’s definition, JE and KS discussed their different interpretations to reach a consensus.

### Phase 2

In the second phase, we analysed researchers’ responses to Question QA14-1 in relation to the different *stages of research*, as defined by the boxes they ticked. For example, we looked at how many researchers had ticked the box to indicate involvement at the ‘design’ stage, and then analysed how often their free-text response matched INVOLVE’s definition of involvement, based on the analysis in phase 1. We did this for all five stages – design, management, undertaking, analysis and dissemination.

We then reviewed the free-text responses for each stage of research where there was a report of involvement reflecting INVOLVE’s definition. For example, we looked at all the free-text responses where the researcher had ticked the ‘design’ box and involvement had been confirmed (*n* = 829). We developed themes based on the different ways the researchers were describing involvement at this stage and produced a summary statement to describe each theme (see Appendix 1). This allowed us to categorise researchers’ responses and quantify how many responses fell in each category. We did this for all five stages.

We also looked at the free-text responses where the researchers had ticked one or more boxes, but where their free-text response did not match INVOLVE’s definition (*n* = 742). We looked for common themes across all five stages, as well as within each stage.

Finally we looked at the free-text responses from researchers who had ticked the ‘none of the above’ box in Question QA14-1, which indicated they had no prior involvement, nor any plans for involvement (*n* = 1017). We reviewed these responses to look for any consistent themes in the justifications given for no involvement. However, many researchers who ticked this box did not provide any further information in the free-text box.

Throughout all of this phase, KS led on the initial analysis and discussed and agreed the themes and conclusions with JE. JE had previously read over half the responses in phase 1 of the analysis, and was therefore familiar with the data.

After completing this analysis, we reflected on how well the outcome met with our expectations. We had clear expectations of the kinds of involvement that might be described at each stage of research, and the impacts likely to be reported, based on our combined experience of many years of involvement practice, INVOLVE’s guidance on involvement [12] and published literature reviews of the impact of involvement [2, 3, 13]. We also reflected on the implications for ethical review, drawing on our experience of the work of RECs and our familiarity with the literature reporting impacts at this stage [3]. JE is currently the Public Involvement Lead at the HRA and KS and was previously a lay REC member.

## Results and discussion

In this section, we have combined the findings from both phases of the analysis to explore how well researchers’ reports of involvement are informing ethical review. Anonymised quotes from researchers’ reports of involvement are included in italic.

### Overall findings

In 2014, 63% of IRAS applicants (total number = 2748) reported involvement in at least one stage of their research study (i.e. ticked one or more of the five boxes on the form, see Fig. 1) [10]. This mirrors the findings from 2010 (62%) and 2012 (61%) [8, 9]. However, analysis of the free text responses revealed that public involvement was confirmed as reflecting INVOLVE’s definition in only 36% of the total number of these studies from 2014. This mismatch suggests there is still some misunderstanding amongst researchers as to what involvement means. Analysis of the reports of researchers who ticked one box or more, but whose reports did ***not*** reflect INVOLVE’s definition were most often describing participation in research (public as subjects of research) or communication of their research to the public or participants (public as recipients of information). However, these responses were very varied.

The most significant finding from the qualitative analysis is that there is a great deal of variation in the way researchers report involvement. Their reports of prior involvement rarely describe involvement in sufficient detail to understand precisely what took place and what difference the involvement made. This has limited our understanding of what researchers may or may not have done around involvement. Therefore REC members, who rely on these written reports, will also be limited in their understanding of the public involvement. For example, researchers often make simple statements along the lines of ‘*Patients and carers were involved in the design of this study*’. This makes it difficult to judge whether the involvement has shaped the study design in any way that would make it more ethically acceptable. When researchers do provide some detail, most often they describe the **method** used, for example ‘*We have discussed this study with X Advisory Group’* or the **task** carried out, for example ‘*An early draft of the study protocol was reviewed by the patient panel’*. Based on this information alone, it is impossible to assess whether such involvement was tokenistic or meaningful, and whether and how it influenced the researchers’ plans. This means that while the free-text descriptions of involvement did report that involvement had taken place, they rarely included the detailed information that could usefully inform a REC’s review.

**Fig. 2:**
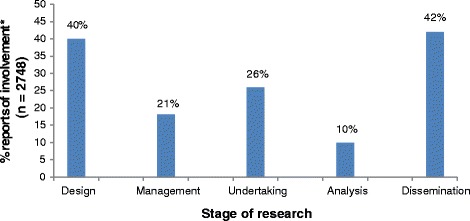
The percentage of researchers who reported involvement at different stages of their research by ticking one or more of the boxes in question QA14-1 on the IRAS form. *Some applicants reported involvement at more than one stage, so the total of the percentages adds up to more than 100%

**Fig. 3:**
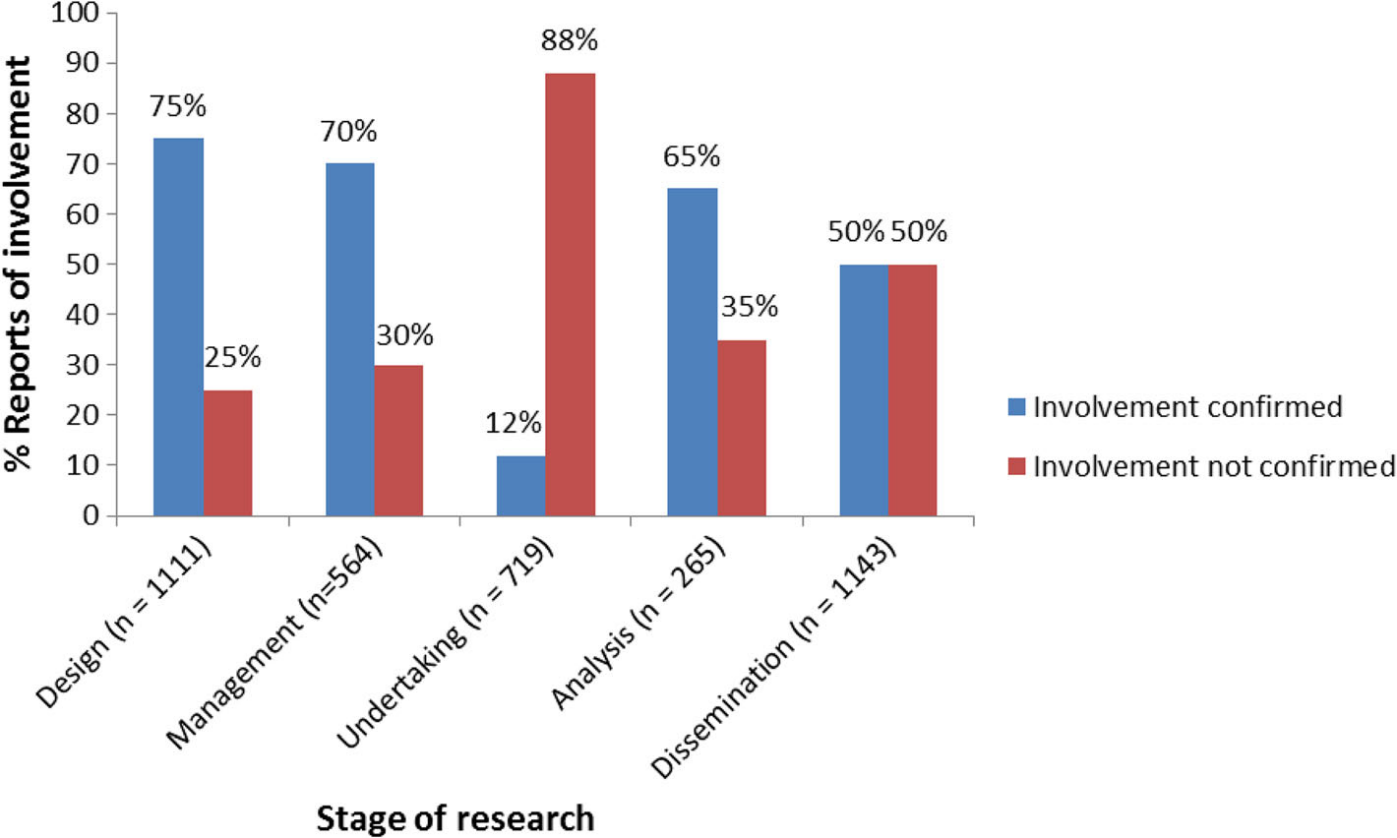
The percentage of researchers’ reports of involvement at each research stage, which either did or did not reflect INVOLVE’s definition of involvement

In the remainder of this section, we report our analysis of how researchers’ free-text responses linked with the stages of involvement listed in question QA14-1: design, management, undertaking, analysis and dissemination. Figure 2 shows how many researchers ticked a box to report involvement at the different stages of research. Figure 3 shows how often their free-text response reflected INVOLVE’s definition of involvement at each of the different stages.

## Involvement in design

If meaningful and high quality involvement has taken place during the design of a study, RECs would be able to draw on researchers’ reports to assure themselves that [3]:

- any information for potential participants has been reviewed to ensure it is easy to understand and meets their information needs
- patients/the public believe the study to be worthwhile
- the study design is ethically acceptable to potential participants and any risks and burdens have been minimised where possible

Eight hundred and twenty-nine researchers described involvement at this stage (see Figs. 2 and 3). Sixty percent of these researchers simply reported that the public had been involved in developing the patient information, for example, ‘*User representatives have reviewed and provided feedback on the patient information sheets’*. However, RECs could not be certain of the nature of the feedback or whether anything had changed in response, as this was rarely described. In nearly half of the 829 studies, involvement in the review of patient information was the only involvement that was reported.

A much smaller proportion (12%) reported involvement in the conceptual design of a project (i.e. involvement in formulating the research question, deciding outcome measures, determining interview questions/focus group schedules and/or developing the intervention). Five researchers (<1%) mentioned that their research project reflected one of the top ten priorities identified via a James Lind Alliance Priority Setting Partnership [14]. Some researchers reported that presentations of their research proposals had met with enthusiasm from patient groups, but this often seemed to reflect one-way communication, rather than partnership working with the public, for example ‘*I have conducted a small PPI group discussion which essentially confirmed… enthusiasm from the group for new treatments*’.

Only 8% of the 829 researchers reported asking the public about ethical aspects of the design such as the best times to ask for informed consent in distressing or difficult circumstances, for example:

‘*This lay group has been particularly helpful in dealing with the ethical issues relating to consent and information sharing… which occur when completing research in incapacitated adults, many of whom do not survive*’.

This is one of the impacts of involvement likely to be most relevant to ethical review.

A larger number (20%) reported that involvement had helped with practical aspects of the study design, particularly in making it easier for people to participate in the research. The public had commented on the length of a questionnaire or the timing of follow-up appointments, for example:

‘*As a result of this feedback we included the costs of drafting in security to allow us to open the … test site at the weekend, to make it easier for people to fit in visits around work or other commitments*’.

However, sometimes this input seemed to be couched in terms of improving the feasibility of the study i.e. increasing the likelihood of capturing the data required, rather than minimising the burden to participants, for example:

‘*They have commented on the proposal during its development and specifically on issues of recruitment of patients, and the feasibility of patient data collection processes*’.

All of these different reports of involvement impacting on the conceptual, ethical or practical design, were limited in detail and only 1% (*n* = 10) described what had changed as a result of the involvement (see Table 1). Most researchers reported having consulted patients (see Table 2 for the range of processes involved), and emphasised the credentials of the group or named individual who had been consulted, seeming to suggest this was an indicator of the quality of the process. However, without details of what questions had been asked and the responses of those consulted, it is impossible to judge whether the involvement had been meaningful. In the vast majority of cases, it would therefore be difficult for RECs to be certain that patients/the public had identified the research topic as a priority and/or had shaped the research in such a way to ensure that the public’s interests and concerns had been appropriately addressed.

**Table 1:** The information provided by researchers who described the impact of involvement at the design stage, in ways that could usefully inform ethical review

In the ten examples of high quality descriptions of involvement, the researchers reported on some or all of the following:
• how patients shaped the research question or why patients thought the research important (not only stating that patients thought it important)
• how patients shaped the intervention and decided which outcome measures to use in clinical trials
• how patients’ input was used to minimise the burden on participants
• how patients influenced the ethical design of a trial – e.g. whether use of placebo would be acceptable
• where patients identified that participants might potentially experience distress and what appropriate changes had been made in response
• how practical arrangements were changed to better meet the needs of participants e.g. follow-up clinics in the evenings and at weekends
• how recruitment processes were changed to be sensitive to the emotional and practical needs of potential participants
• how patients were involved in deciding what questions to ask in interviews/ focus groups, rather than only being asked comment on the wording of questions written by researchers
• what questions patients were asked in reviewing the protocol and patient information, the responses they gave and the changes made as a result
• how patients would continue to be involved in the project at different stages, with a clear explanation of what input was expected and how it might shape future decisions
It is of note that no researcher discussed potential ethical concerns raised by involvement in data collection and analysis, which is a key aspect of the ethical review of involvement.

**Table 2:** The range of approaches used to consult the public during the design of a study that were confirmed as involvement

• Clinicians talking to a few patients in their clinic
• Presenting the proposal at an event or conference
• Presentations to patient groups – either local groups or linked to a charity
• Consulting standing public involvement panels within local trusts or universities, research networks, charities
• Discussion with one named patient
• Organising a meeting with patients (sometimes with National Institute for Health Research (NIHR) Research Design Service funding)
• Setting up an advisory group for the project for consultation at this stage and subsequently deciding to keep the group in place for the remainder of the project
• Consulting a number of patient groups including project specific and professional groups
• Consulting a steering committee or advisory group overseeing the project which had patient members as well as professionals
• Developing the study within a Network Clinical Studies Group
• Receiving input via patient members of the funders’ grant review committee

We were surprised to find that nearly a fifth (18%) of researchers who described involvement in the design of their study were planning to do this *after* the REC review. For example one researcher stated, *“A service-users group will be consulted to seek their views on whether or not it is appropriate to seek consent from the relatives of a deceased child”*, an important ethical issue that RECs would be concerned about. This shows a lack of understanding of how involvement can improve the ethical acceptability of research and inform the ethical review process. There are also practical implications for these researchers, who may need to resubmit revised protocols and information sheets following involvement, potentially creating delays in getting their research started. This also has implications for the workload of the RECs.

Although involvement at the development stage may raise ethical issues for researchers to consider, it has been recommended that these be addressed voluntarily in a self-regulatory way, rather than requiring REC review [15, 16]. The ethical issues for RECs raised by involvement at other research stages are discussed below.

## Involvement in management

Four hundred and eight applicants described involvement at this stage, but in very different ways. Our expectations of involvement in the management of research is that members of the public are in some way involved in making decisions about the course of a project *in real time*. For example, they might influence decisions about recruitment, in response to problems with recruitment rates. REC members might wish to be reassured that such plans for involvement will enable the interests of potential participants to continue to influence decision-making within the project. They may also want to assess whether any ethical issues are raised by the involvement of Steering Group members in data analysis [15] (see below).

80% of the researchers did describe plans for this kind of involvement, via a project-specific advisory group, membership of a steering or management group, or ongoing consultation of an external patient group, for example ‘*The Steering Group members include two adult survivors of childhood cancer*’. However, most applicants described the process rather than the purpose of this involvement. They tended to describe how often the group would meet over the course of the project, rather than identifying how they hoped this involvement would make a difference. Such involvement could be meaningful or tokenistic, depending on how well such an oversight group is chaired, trained and supported [17]. Additional information might therefore be required for REC members to feel assured that this kind of involvement will be effective.

By way of contrast, nearly a fifth of researchers who reported they were involving patients in the management of their study did not mention any form of ongoing involvement. Instead they described some form of consultation that had already taken place, for example, ‘*Two service user researchers have been involved in planning the research*’. This finding suggests that these researchers understood that if they had consulted the public during the design stage, and that the consultation had changed some aspect about how they planned to run the project, then this would constitute ‘involvement in managing the project’. This suggests that some researchers may misunderstand what this type of involvement requires.

## Involvement in undertaking research

This is one of the few areas where involvement in research may raise ethical concerns for RECs [15], particularly where the public are involved in collecting and analysing data (conducting interviews, facilitating focus groups and/or recruiting participants). Consideration must then be given to the well-being and safety of the people who are actively involved as researchers as well as the well-being, safety and preferences of the people who are taking part in the research as study participants [15].

Although a large number of researchers (*n* = 719) reported involving the public at this stage, only 12% of them were actively involving the public as co-researchers as described above. In these cases, the researchers often reported that they were following INVOLVE’s good practice guidelines in training and supporting these individuals and paying for their time. However, no researcher made explicit mention of any ethical issues that might be raised by this involvement or how these might be addressed.

The remaining 88% of researchers who reported involvement at this stage, often went on to describe some other kind of involvement, including:

- a previous consultation exercise, for example, ‘*Discussions were had with patients on the best methods of data collection for the study team and the patient, and follow-up telephone interviews seemed most appropriate*’
- involvement in an oversight group, for example, ‘*A patient and a carer will be asked to join the Advisory Group for the study and will be involved in discussions as the research progresses*’

It seems that if an early consultation led to a change in the way researchers planned to conduct their project, (or researchers planned to consult an oversight group in future), then this was understood to mean that involvement had made (or would make) an impact on the ‘undertaking’ of the research. This again highlights a common misunderstanding about what active involvement looks like at this stage.

## Involvement in analysis

Of the 265 applicants who reported involvement at this stage, 68% described involvement as defined by INVOLVE. In line with our expectations, some described how the public would be expected to provide an alternative perspective on the results, for example:

‘*They will be involved in the analysis of results, in particular, we will be interested to note whether the data from research is interpreted differently by the patient and public compared to the researchers*’.

Others reported that they would ask patients to interpret the findings i.e. to reflect on the researchers’ analysis to draw out the implications for the patient community and/or for health service policy and practice, for example:

‘*Service users, carers and professionals will be involved in the analysis of results through focus groups designed to validate the findings and explore the outcomes for further research and clinical implementation*’.

In some cases, it was expected that an oversight committee with public members would be involved in this exercise, for example:

‘*The design, management, undertaking, analysis and dissemination of results comes under the remit and jurisdiction of the Trial Steering Committee. This committee has a lay member who is a breast cancer survivor*’.

Such active involvement of the public in analysing data may raise ethical concerns, particularly in relation to maintaining the confidentiality of patients’ data [15]. None of the researchers reporting plans for involvement at this stage made explicit reference to the ethical issues that might arise or how these would be managed.

## Involvement in dissemination

In support of greater transparency in research, and in order to promote the interests of participants, the REC review aims to ensure that researchers share the results of their research with the people who took part and the wider patient community [18]. Our expectations are that active involvement of the public in dissemination would involve members of the public in writing or deciding the content of any reports of the findings, and/or presenting the results [19, 20]. This helps to ensure the reports are easy to understand and contain the information that most interests patients and the public. Involvement in presenting the findings is reported to have greater power and influence than when researchers report the findings alone [19]. However, only 16% of the 571 researchers who reported involvement at this stage described these kinds of approaches, for example:

‘*We are also working with patients to disseminate findings of the research for example we are running a workshop jointly with a stroke survivor*’.

More commonly, researchers planned to make use of the involved individual’s connections with patient groups/organisations to disseminate the results to the wider community, for example, ‘*Dissemination of our results to the wider [patient] population will be through the [X] charity*’. While making use of personal networks is an important added-value of involvement [20], the potential impact of involvement is limited if this is all that is done.

Some applicants reported involvement at this stage, even though they only intended to communicate their findings to participants or to publish their findings on a website, or in a newsletter or journal, for example, ‘*All study outputs will be disseminated via the [*local patient group*] website*’. On this basis, REC members might be assured that a report of the results will be disseminated to the wider community, but without being assured of meaningful involvement, they may not always be certain that such reports will be accessible and relevant to patients/the public [5].

## The rationale given for no involvement

Not all researchers gave a rationale for *not* involving the public in their research, often giving no response at all. The range of reasons that were given are listed in Table 3. These were very varied and therefore we have not attempted to quantify the frequency of different responses. Some of these seemed justifiable for example, not being able to find people affected by extremely rare diseases (where there may only be 1 or 2 people affected in the country). Others reflected common misperceptions about involvement, such as the public not being able to contribute technical knowledge, not being able to involve children in research or there being no value in involving the public in basic research. Sometimes researchers simply commented that ‘*involvement was not necessary or appropriate*’ for their study, without further explanation. This is again an area where researchers will need to provide more detail for RECs to be able to make a proper assessment of whether involvement is needed and could usefully inform their review.

**Table 3:** Examples of justifications given for not involving the public in research

• Experienced professionals (clinicians and researchers) have already developed the study design
• Commercial sensitivities in relation to clinical trials
• Phase 1 or 2 trials with healthy volunteers, and little room to influence study design
• Concerns about access to confidential data
• Highly technical nature of the research means that patients/ the public would lack the knowledge/ skills required
• Lack of resources
• Research involves basic science or minimal participation of patients e.g. use of a biopsy sample
• Challenges of finding patients to involve (e.g. people infected by extremely rare conditions)
• Challenges of involving children in research
• Little room for patients to influence the design of the study e.g. comparison of two technical measures
• The study was designed outside of the UK so no involvement of UK patients
• The study is a pilot study or proof of concept study
• The responsibility for the design of the study lies with the sponsor
• The study does not require any deviation from routine clinical practice
• The study is using the same approach as a previous study and therefore no new issues for patients to consider
• Involvement is not necessary or appropriate for the study (without stating why)

## Conclusions

Public involvement in the design stage of research, *prior* to ethical review, has the potential to increase the ethical acceptability of the research, and to facilitate the decision-making process for RECs [3, 4, 21]. This review of the researchers’ reports of involvement in the IRAS application forms, suggests that many researchers may still be unclear about this particular added-value of involvement. The contributions of members of the public towards improving the ethical acceptability of research are very rarely made explicit in researchers’ reports. The information that researchers do provide more often describes the method that was used or the task that was completed, rather than the difference the involvement made. This makes it difficult for REC members to feel confident that the opinions of patients/the public have meaningfully influenced the research design and addressed any issues of ethical concern.

REC members also have an interest in researchers’ plans for involvement in the future delivery of a research study that follows *after* the review process. This is not to judge the quality of the researchers’ proposals for involvement (which might well need to be undertaken by other parts the research regulatory system), but to be assured that participants’ interests and concerns will continue to influence the researchers’ conduct, particularly in relation to sharing the findings with participants and other patients. They will also want to check that any ethical issues raised by involving the public in collecting and analysing data have been considered and appropriately addressed. Again, the information currently being provided by researchers is not enabling REC members to make this kind of assessment. Not only is there a general lack of clarity about what active involvement means at these latter stages, but the ethical issues are rarely being considered.

### Implications for the HRA

This analysis reveals that very few researchers report anything about involvement that could assure RECs and help to inform the ethical review of applications. Furthermore, the variation in what researchers report about involvement at each stage of research indicates that they are not clear what the question on IRAS is asking. The question itself and the accompanying guidance may be contributing to this confusion. Therefore, the HRA has decided to work with RECs and the research community (researchers, funders and sponsors from both non-commercial and commercial sectors), to determine what information about involvement, its impact in the early design stage of research and its implementation in later stages, would facilitate the REC decision-making process [10]. This work will be used to replace the question about public involvement in the IRAS in 2018 to ensure that researchers provide the information that REC members need. At the same time the HRA will develop guidance and training for REC members and the research community about how involvement can potentially improve the ethical acceptability of studies, which might lead to more applications being approved outright first time.

### Implications for practice

The researchers’ responses to the QA14-1 question also reveal where there is still considerable misunderstanding around when and where to involve the public in research, which may be limiting its potential. By far the greatest majority of researchers seem to understand that involvement has largely a communications function, to support the production of information for participants in plain English, during recruitment and in the dissemination of findings. There is far less recognition that the public usefully contribute to the conceptual, practical and ethical aspects of research design and that active involvement in dissemination helps to tailor the information to target audiences and increase the power of that communication. But perhaps the most common misunderstanding is the difference between active involvement in the conduct of research and involvement in groups with an oversight or advisory function, which may differ in the extent to which the public contribute to data collection and analysis. These areas might benefit from further discussion and elaboration in future training and guidance for researchers. Moreover, training and guidance could constructively challenge some of the common misperceptions around why involvement is not possible or desirable in certain kinds of research.

## Appendix 1

**Table 4:** A list of the themes and subthemes that were developed from the data and used to code the free-text responses in researchers’ response to question QA14-1 of the IRAS form

Theme	Sub-theme
Involvement in design	Public were consulted Public member of planning team Review of plans by committee/ steering group Participants gave feedback at early stage Protocol was reviewed Patient information sheet was reviewed Topic confirmed as important Impact on conceptual design Impact on practical design Impact on ethical design Impact on recruitment strategy Impact on design of intervention Simple statement reporting involvement at this stage No involvement matching INVOLVE’s definition
Involvement in management	Consultation of pre-existing patient group Project advisory group Project management group Steering Group/ Committee Trial management group Simple statement reporting involvement at this stage No involvement matching INVOLVE’s definition
Involvement in undertaking	Participation in research Participants giving feedback to researchers during project Giving feedback to participants/ the public on progress Involved in delivering training as the intervention Involved as interviewers Involved in recruitment Involved as service user researchers Members of Steering Group with oversight Simple statement reporting involvement at this stage No involvement matching INVOLVE’s definition
Involvement in analysis	Focus group to discuss findings Public to comment on implications for practice Involvement in analysing data Findings shared with Steering Group Simple statement reporting involvement at this stage No involvement matching INVOLVE’s definition
Involvement in dissemination	Dissemination to clinicians Dissemination to patients Dissemination to the participants Working with a patient organisation to disseminate findings Taking part in public engagement events Public give talks/ presentations Public write or co-author reports and publications Public comment on researchers’ final reports Steering Group comment on researchers’ final report Simple statement reporting involvement at this stage No involvement matching INVOLVE’s definition
Timing of involvement	Planned for the future after ethical review
Good practice	Good practice [consistent with INVOLVE’s values and principles] described
Reasons for no involvement	Benefits of research are evident A clinician already gave advice on the project Commercial trial Confidentiality concerns Participants are healthy volunteers Project highly technical Job of sponsor to comment on project No resources for involvement Project has minimal patient participation No patients available No room for influence This is a pilot study Study involved routine clinical practice Project is the same design as previous project Simple statement = not required or appropriate

## Abbreviations

HRA: Health Research Authority
IRAS: Integrated Research Application System
NIHR: National Institute for Health Research
REC: Research Ethics Committee
RES: Research Ethics Service

## Footnotes

1 We use the term ‘public’ to include, patients, potential patients, carers and people who use health and social care services as well as people from organisations that represent people who use services, in line with INVOLVE’s definition of public involvement [22].

2 The HRA’s terms and conditions in relation to the protection of data in the IRAS forms, state that identifiable information cannot be used, nor can this information be supplied to a third party. The use of anonymised quotes from researchers’ reports of involvement for research purposes is therefore in line with this agreement. KS was also required to sign a confidentiality agreement with the HRA prior to working on this project.

3 ‘Non-educational’ studies are externally funded research studies. They are distinct from ‘educational studies’ where the principal purpose is training researchers, for example, in completing doctoral or masters research degrees. In 2014 there were 4074 applications to NRES. After removal of records with no data for the public involvement question there were 2748 non-educational records and 1228 educational records.

4 INVOLVE defines public involvement in research as research being carried out ‘with’ or ‘by’ members of the public rather than ‘to’, ‘about’ or ‘for’ them [22].
